# Silver nanoparticles in complex media: an easy procedure to discriminate between metallic silver nanoparticles, reprecipitated silver chloride, and dissolved silver species

**DOI:** 10.1039/c8ra04500c

**Published:** 2018-07-05

**Authors:** Kateryna Loza, Matthias Epple

**Affiliations:** Inorganic Chemistry and Center for Nanointegration Duisburg-Essen (CeNIDE), University of Duisburg-Essen Universitaetsstr. 5-7 45117 Essen Germany matthias.epple@uni-due.de

## Abstract

Silver nanoparticles undergo oxidative dissolution in water upon storage. This occurs in pure water as well as in more complex media, including natural environments, biological tissues, and cell culture media. However, the dissolution leads to the reprecipitation of silver chloride as chloride is present in almost all relevant environments. The discrimination between dissolved silver species (ions and silver complexes) and dispersed (solid) species does not take this into account because all solid species (metallic silver and silver chloride) are isolated together. By applying a chemical separation procedure, we show that it is possible to quantify silver, silver chloride, and dissolved silver species after immersion into a typical cell culture medium (DMEM + 10% FCS). During the dissolution of metallic silver nanoparticles, about half of the dissolved silver is reprecipitated as solid silver chloride, *i.e.* the mere analysis of the soluble silver species does not reflect the true situation. The separation protocol is suitable for all chloride-containing media in the presence or in the absence of biomolecules.

## Abstract 2

Dissolved ionic silver species and reprecipitated silver chloride are quantified as products of the oxidative dissolution of silver nanoparticles.

## Graphical abstract

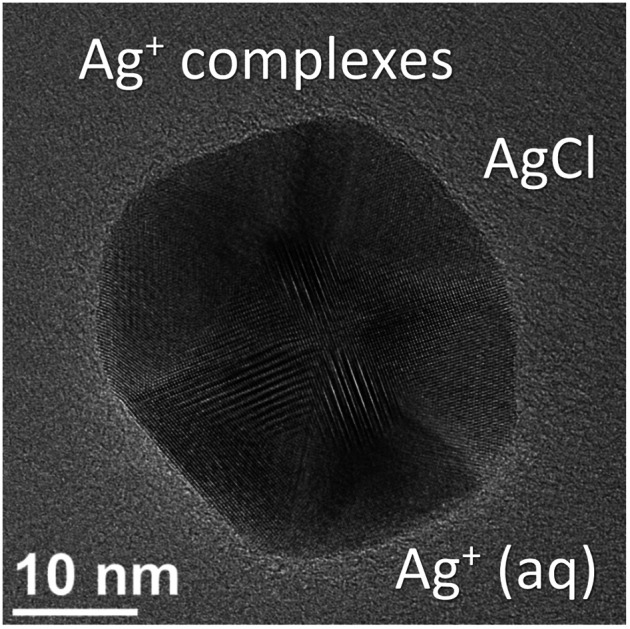

## Introduction

Silver nanoparticles are widely used due to their antibacterial action in consumer products and in medicine.^1–10^ This has resulted in growing concerns about possible risks for humans and the environment.^3,11–18^ However, in the majority of cases silver nanoparticles show an indirect action, acting as a depot for silver ions that are responsible for biocidal action.^19^ The release of silver ions from silver nanoparticles occurs after oxidation. Under normal conditions, the oxidative agent is dissolved oxygen, as has been demonstrated by several authors.^19–27^ This is also the case for biological media where no oxidizing species like H_2_O_2_ are present. Ho *et al.* have presented a model on the dissolution of silver nanoparticles in the presence of oxygen.^25,27^ Notably, silver nanoparticles do not dissolve in the absence of dissolved oxygen^20^ and there is also no bactericidal effect in this case.^19^ The ion release is proportional to the specific silver surface area for differently shaped silver nanoparticles.^22^

Studies on the dissolution behaviour of silver nanoparticles in various aqueous media were reported,^9,19,21,22,25,27–35^ but questions remain on the dissolution behaviour in more complex media, *e.g.* in biological systems and in the environment.^20,36^ The surface functionalization of the silver nanoparticles was variable, but the effect of the colloidally stabilizing shell is difficult to assess (see ref. 20 for a literature review). It was found that the presence of cysteine strongly reduces the release of silver, probably by surface passivation.^20^ Despite the fact that they are complicated in composition and nature, biological media are the relevant conditions when it comes to an assessment of the potential risk of silver, *e.g.* in the body.

It has been shown that the predominant species of silver ions interacting with cell culture components is nanoparticulate silver chloride. Silver chloride forms rapidly when silver nanoparticles or silver ions come into contact with chloride-containing media.^33,37,38^ However, the commonly used separation techniques (centrifugation, nanofiltration, dialysis) are unable to differentiate between different solid (nanoparticulate) silver species, therefore a chemical separation step is necessary. The methods for the separation of ions and nanoparticles in biological tissues have been reviewed by Su *et al.*^39^

Here, we present a straightforward protocol for the determination of silver chloride besides silver nanoparticles and soluble silver species after immersion of silver nanoparticles into chloride-containing media.

## Experimental section

### Synthesis of silver nanoparticles

Poly(vinylpyrrolidone) (PVP)-coated silver nanoparticles were synthesized by reduction of silver ions with glucose in the presence of PVP in water according to Wang *et al.*^40^ 2 g glucose (11.10 mmol) and 1 g PVP (0.025 mmol based on the molar mass of the monomer *M*_w_ = 40 000 g mol^−1^) were dissolved in 40 g water and heated to 90 °C for 30 min. Then, 0.5 g AgNO_3_ (2.94 mmol) dissolved in 1 mL water was quickly added. Before to the addition, the silver nitrate solution was kept under strict light exclusion to avoid photoreduction. It has been shown that the presence of nuclei from photoreduction in silver nitrate may influence the nanoparticle synthesis.^41,42^ The dispersion was kept at 90 °C for 1 h and then allowed to cool to room temperature. The synthesis was carried out under continuous vigorous stirring. During the reaction, the color of the solution changed from transparent pale yellow to opaque grey. The particles were collected by ultracentrifugation (29 400 g; 20 000 rpm; 30 min), redispersed in pure water and collected again by ultracentrifugation. In that way, NO_3_^−^, excess glucose and its oxidation products, excess polymer, and excess Ag^+^ were all removed. Then, the silver nanoparticles were redispersed in pure water by ultrasonication. Degassed and argon-saturated ultrapure water was used in order to avoid an oxidation by dissolved oxygen and a subsequent release of silver ions from the particles during storage. All samples were stored at 4 °C in the dark until further use. The nanoparticles were characterized by scanning and transmission electron microscopy, and dynamic light scattering. The final silver concentration in all dispersions was determined by atomic absorption spectroscopy.

### Characterization of silver nanoparticles

Scanning electron microscopy (SEM) was performed with a FEI Quanta 400 ESEM instrument in high vacuum. Transmission electron microscopy (TEM) was carried out with an aberration-corrected FEI Titan transmission electron microscope equipped with Cs-probe corrector (CEOS Company), operated at 300 kV.^43^ The hydrodynamic diameter and the zeta potential of the nanoparticles were measured by dynamic light scattering (DLS) using a Malvern Zetasizer Nano ZS. The polydispersity index (PDI) was below 0.3 in all cases.

### Analysis of silver nanoparticle dissolution in DMEM supplemented with 10% FCS by atomic absorption spectroscopy

Silver nanoparticles were added to Dulbecco's modified Eagle's medium (DMEM) supplemented with 10% fetal calf serum (FCS) at a silver concentration 0.05 g L^−1^ (50 ppm) to a total volume of 25 mL. The dispersions of silver nanoparticles in medium were incubated at 37 °C with gentle shaking for various time periods under sterile conditions. After incubation, the samples were ultracentrifuged (77 000 g; 35 000 rpm; 30 min) and the supernatant, containing all soluble silver species, was pipetted off as far as possible without removing the solid residue. 2 mL of the supernatant were mixed with 2 mL NH_3_ (32%) and 2 mL H_2_O. The residue, containing silver nanoparticles and silver chloride, was washed three times with ultrapure water by repeated ultracentrifugation (77 000 g; 35 000 rpm; 30 min). Then the residue was mixed first with 1 mL of concentrated HNO_3_ (65%) and then 4 mL ultrapure water to dissolve metallic silver nanoparticles, shaken well, and centrifuged (1250 g; 4000 rpm; 60 min). The supernatant (containing the former silver nanoparticles, dissolved by HNO_3_) was diluted with 10 mL ultrapure water. The new residue (containing silver chloride) was treated with 1 mL of H_2_O and 2 mL NH_3_ (32%), shaken well, and diluted with 10 mL ultrapure water. This led to the dissolution of silver chloride to the diammine silver(i) complex.

From incubation until the last step, the samples were treated and kept in the same thick-walled 10 mL centrifuge tubes to avoid losses. To achieve a reproducible and consistent recovery for all samples, an optimization of the analytical process was necessary. First, it turned out that in spite of strongly basic or acidic solutions, adsorption of silver compounds took place on the vessel walls of the polypropylene tube. To remove this error, all samples were treated by ultrasonication before the separation steps.

The concentration of silver was determined by atomic absorption spectroscopy (AAS; Thermo Electron Corporation, M-Series). The detection limit was 1 μg L^−1^ (1 ppb).

All dissolution experiments were performed at least in duplicate. The values were averaged.

## Results and discussion

**Fig. 1:**
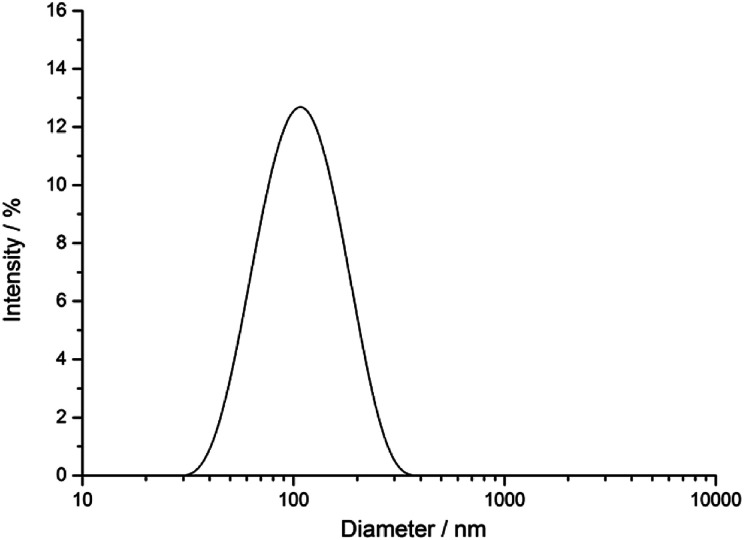
Characterization of PVP-coated silver nanoparticles by dynamic light scattering.

The silver nanoparticles were characterized by scanning electron microscopy and dynamic light scattering. All measurements were performed with the purified nanoparticles. The hydrodynamic diameter as measured by dynamic light scattering was about 120 nm (Fig. 1). The hydrodynamic diameter gives information about the metal core including the outer ligand shell that consists of adsorbed PVP in our case. The polydispersity index (PDI) was lower than 0.3 in all cases, *i.e.* it is typical for a well-dispersed system.

**Fig. 2:**
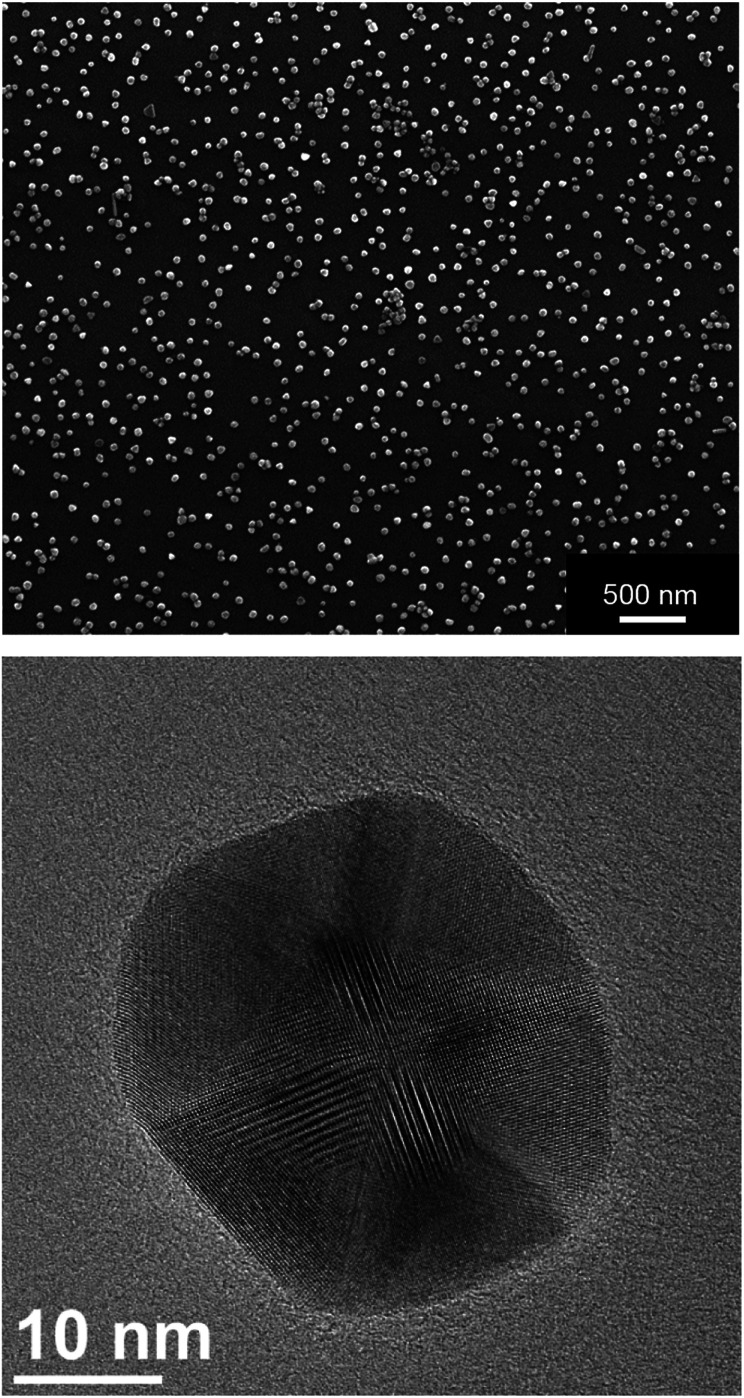
Scanning electron micrograph (top) and transmission electron micrograph (bottom) of silver nanoparticles. Note the facetted nature of the nanoparticle in the TEM image.

Fig. 2 shows typical SEM and TEM images of the silver nanoparticles. For SEM analysis, the diluted dispersion of purified silver nanoparticles was dried on a silicon wafer. For TEM analysis, the diluted dispersion was drop-cast onto a carbon-coated copper grid and dried under ambient conditions. The particles were well distributed on the substrate and showed no strong agglomeration and a uniform size distribution. The average diameter of the metallic core was about 65 nm.

Based on our earlier results on the dissolution of dispersed silver nanoparticles in pure water, physiological NaCl solution, phosphate buffered saline (PBS), and cysteine,^20,22,31^ the studies were extended to a more complex medium, *i.e.* cell culture medium supplemented with protein-rich fetal calf serum (FCS). The dissolution kinetics of the silver nanoparticles were analysed after immersion in Dulbecco's modified Eagle's medium (DMEM) supplemented with 10% FCS at 37 °C. DMEM is a common cell culture medium for eukaryotic cells and tissue. It contains salts, amino acids, vitamins, and glucose.^44^ The chloride concentration in the cell culture medium is 119 mM.^33^ FCS is often used to maintain cell growth.^45^

**Fig. 3:**
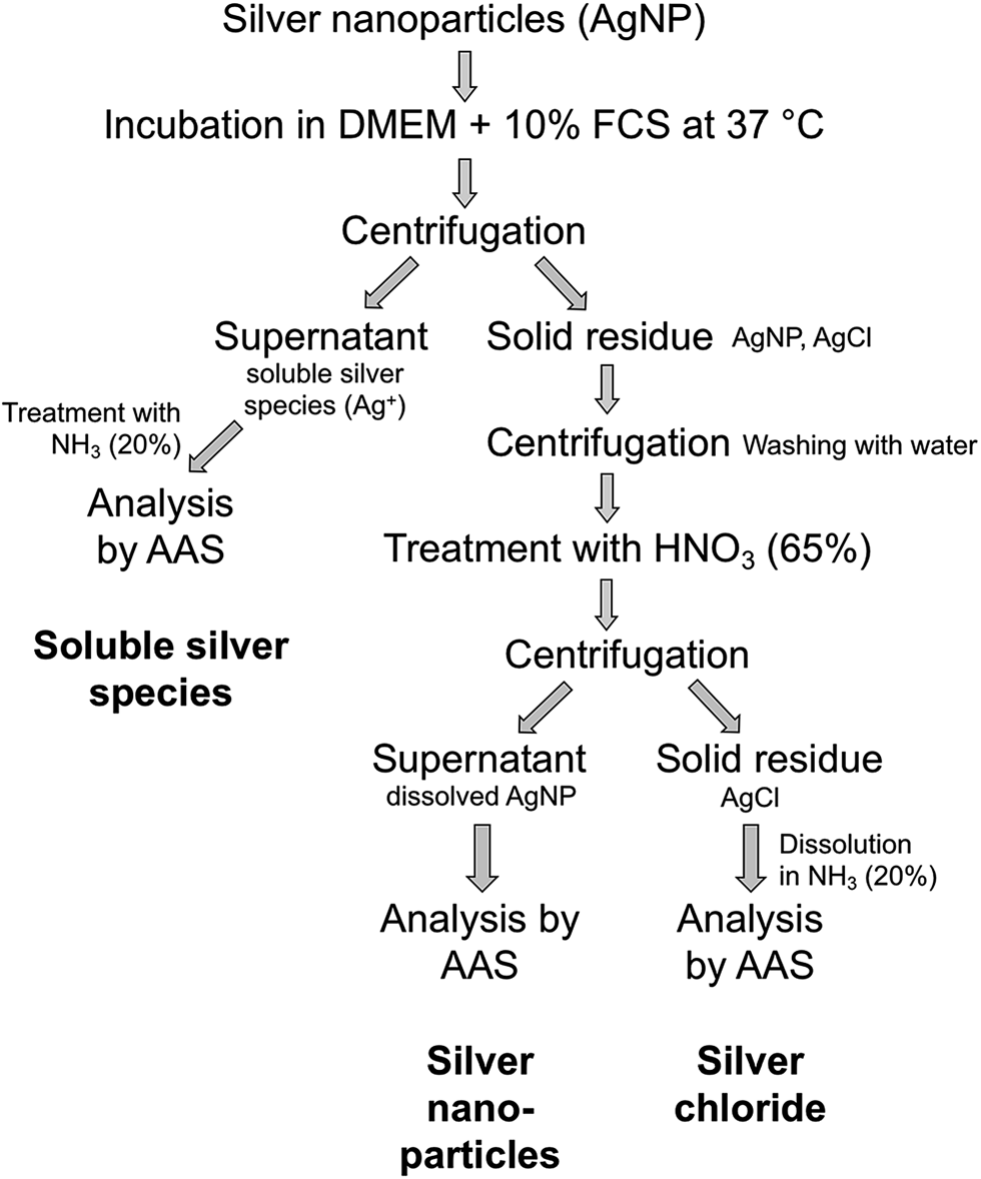
Schematic representation of the protocol for the quantification of silver nanoparticle dissolution.

Ultracentrifugation was used as an efficient method for the separation of solid compounds from all dissolved species. After certain periods, dispersions of possible silver chlorido- or protein-complexes were separated from nanoparticulate silver chloride and remaining silver nanoparticles by ultracentrifugation. The precipitate, consisting of AgCl particles and Ag nanoparticles, was washed to remove the remaining immersion medium. This is an essential point because residual medium still contains a lot of chloride that would interfere with the subsequent analyses. Afterwards, the dispersion was treated with nitric acid to dissolve the remaining silver nanoparticles for further quantification. The resulting dispersion was centrifuged to isolate the solid silver chloride. Finally, the precipitated silver chloride was treated with ammonia solution to convert it into soluble the silver diamine complex [Ag(NH_3_)_2_]^+^. All solutions were then analysed by atomic absorption spectroscopy (AAS). A schematic representation of the quantification protocol is shown in Fig. 3.

Such a standard procedure permits the assessment of silver nanoparticle dissolution in complex media, including a determination of silver chloride that has resulted from silver nanoparticle dissolution, followed by reprecipitation in contact with chloride.^20,33,37,38^ A validation of each experimental point was possible by computing the sample recovery level in all cases. The recovery level was 100% for all samples within the experimental error (standard deviation of the AAS, losses during the washing process, pipetting errors).

**Fig. 4:**
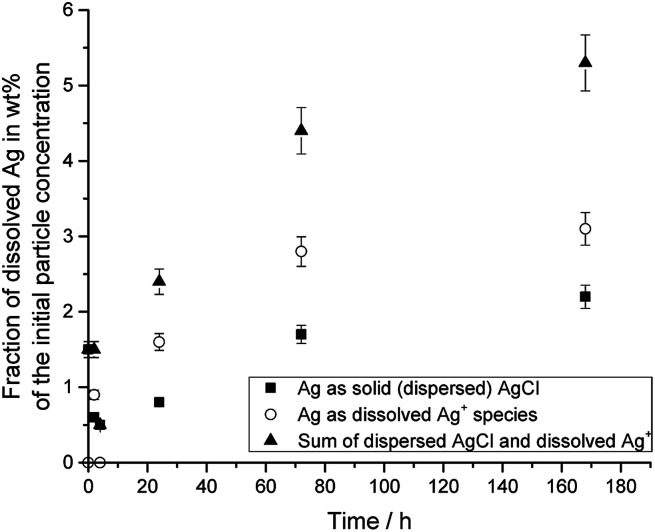
Dissolution of PVP-coated silver nanoparticles immersed in DMEM + 10% FCS at 37 °C. The initial concentration of silver nanoparticles was 50 μg mL^−1^.

Fig. 4 shows the dissolution data for silver nanoparticles in DMEM/10% FCS at 37 °C, *i.e.* under physiological conditions.

The silver nanoparticle dissolution slowly approached a saturation state at about 6%. About 5.3% of silver nanoparticles were dissolved after one week, but 2.2% had re-precipitated in the form of solid silver chloride. The remaining 3.1% were present as dissolved silver compounds in the medium.

The dissolution in DMEM/FCS found here is considerably slower than the dissolution of equivalent silver nanoparticles in water or glucose-containing solution and comparable to the dissolution in 0.9% NaCl solution or in PBS, recorded as free Ag^+^ ions.^20^ It is also slower than the dissolution of silver nanoparticles with different size and shape in water.^22^ This is probably due to a partial blocking/passivation of the silver surface by silver chloride.

The presence and analysis of different silver species in complex media has been addressed in a number of publications.

Tan *et al.* have separated silver ions and citrate-coated silver nanoparticles by hollow fibre flow field-flow fractionation (HF5), followed by ICP-MS. In domestic wastewater, they detected agglomeration and also the formation of silver-sulphur species.^46^

Merrifield *et al.* have studied core–shell Au–Ag nanoparticles in natural water, containing fulvic acid. By single-particle-ICP-MS (SP-ICP-MS), they found that the particles agglomerated, partially dissolved, and that AgCl reprecipitated on the silver nanoparticle surface.^47^

Sirirat *et al.* showed that silver nanoparticles and ionic silver can be distinguished in thermospray flame furnace atomic absorption spectrometry after careful calibration.^48^

Dong *et al.* showed that the dissolved silver can be measured after inducing the agglomeration of the remaining silver nanoparticles by the addition of calcium nitrate (removal of the colloidal stabilization). This underscores the sensitivity of electrostatically stabilized nanoparticles to an increased ion strength.^49^

Zook *et al.* have developed a method to distinguish between silver nanoparticles and silver chloride by UV-Vis absorption spectroscopy. However, this method is not universally applicable as particle agglomeration and scattering by light biomolecules can disturb the quantification.^33^

Su *et al.* have studied the dissolution of PVP-coated silver nanoparticles *in vivo* in different tissues after separating ions from nanoparticles in a PTFE knotted reactor, followed by ICP-MS. They found that the majority of silver nanoparticles had dissolved after a few days.^39^

Hansen and Thünemann have studied the dissolution of PEG- and PVP-coated silver nanoparticles by flow field-flow fractionation, TEM, DLS, SAXS, and ICP-MS. They found that the dissolution was strongly enhanced in cell culture medium (DMEM) after the addition of 10% FCS. The nanoparticle corona consisted mainly of albumin.^50^

Graf *et al.* found a remarkable enhancement of the dissolution rate of silver nanoparticles in aqueous acetate buffer (pH 4) for PVP-coated silver nanoparticles. In contrast, silver nanoprisms dissolved at about the same rate at pH 4 and at pH 7.^21^

Thomas *et al.* have studied the dissolution of citrate-coated silver nanoparticles in RPMI with different amounts of FBS (1, 10, and 30%). Ions and particles were separated by centrifugation. Silver was determined by ICP-MS. The dissolution of the silver nanoparticles was strongly enhanced at increasing levels of FBS. The dissolved fraction was about 20% after 72 h in RPMI + 10% FCS. They also included the dissolution rate in a particokinetic model.^51^

Sikder *et al.* have presented a method to quantify the dissolution of PVP-coated silver nanoparticles in seawater by a combination of UV-Vis spectroscopy, AFM, and ICP-MS after separating ions and nanoparticles by ultrafiltration. They found an almost complete dissolution in seawater after 120 h.^52^

Kaiser *et al.* reported that the dissolution of silver nanoparticles was strongly dependent of the culture media, using a combination of a silver ion-selective electrode and ICP-MS and by taking into account the formation of soluble silver–chlorido complexes. In salt-containing cell culture media, the amounts of dissolved silver (as Ag^+^ and as chloride complex) were generally very low, both after the addition of silver nanoparticles and a water-soluble silver salt. The presence of FCS enhanced the cell viability.^38^

The nature of the dissolved silver species remains open. Besides Ag^+^ (aq) and dissolved chlorido complexes,^38^ it is very likely that silver is also coordinated by the biomolecules present in the cell-culture medium. Chemically, there are many possibilities to bind silver. Although silver binds most strongly to P and S donor ligands, it also has an extensive coordination chemistry with N and (to a lesser extent) O donors, and appears to bind to almost every kind of biomolecule.^53^ Ag^+^ has long been known to form complexes with amino acids.^53^ This is often used to stain proteins in polyacrylamide gels.^54^ In certain proteins, silver has been shown to bind to specific sites, forming Ag_6_, Ag_12_ and Ag_18_ clusters with rabbit metallothionein.^55^

The effects of different nutrition media have been specifically investigated.^56^ Two Ag(i) phosphine complexes showed an antifungal activity that was lost in serum-rich medium. The toxicity of one of these complexes towards tumour cells *in vitro* was reduced to a fifth in the presence of FCS in the medium.^57^ The biological effect of PVP-stabilized silver nanoparticles and silver ions on human mesenchymal stem cells was studied in pure RPMI and in mixtures of RPMI/bovine serum albumin (BSA) and RPMI/FCS, respectively. Both BSA and FCS considerably decreased the cytotoxicity of silver ions and of silver nanoparticles, indicating a binding of silver by these proteins.^58^ These effects were attributed to the reaction with serum and other components of the media.^57,58^

## Conclusions

We have established a protocol to follow the dissolution of silver nanoparticles in chloride-containing complex biological media in more detail. This including a distinction between silver nanoparticles, reprecipitated silver chloride, and all soluble silver species. It shows that the dissolution of silver occurs about twice as fast as expected from the fraction of soluble species, with about one half of the dissolved silver reprecipitating as silver chloride. By a sequential dissolution sequence where first metallic silver is dissolved in nitric acid and then silver chloride is dissolved in concentrated ammonia, accompanied by centrifugation steps, a quantitative analysis is possible. It is important to distinguish between metallic silver and sparingly soluble silver chloride because Ag can only be dissolved by oxidation whereas AgCl can also be dissolved by dilution or in the presence of coordinating agents. Thus, the transformation/dissolution kinetics into soluble silver species are different. The nature of the formed silver chloride is not known, but it is likely that it is present on the surface of the remaining silver nanoparticles as well as individual particle in dispersion.

In general, this method can be also used for silver nanoparticles in different chloride-containing media, in the presence or in the absence of biomolecule.

## Conflicts of interest

There are no conflicts to declare.

## Supplementary Material
